# Immunopharmacology in Vernal Keratoconjunctivitis: Current and Future Perspectives

**DOI:** 10.3390/ph14070658

**Published:** 2021-07-09

**Authors:** DeGaulle I. Chigbu, Bisant A. Labib

**Affiliations:** Pennsylvania College of Optometry at Salus University, 8360 Old York Road, Elkins Park, PA 19027, USA; blabib@salus.edu

**Keywords:** vernal keratoconjunctivitis, clinicopathologic correlations, immunomodulators, immunobiologics, pharmacotherapy

## Abstract

Vernal keratoconjunctivitis (VKC) is a complex and chronic, multifactorial Th2 cell-mediated chronic ocular surface inflammatory condition that typically affects predominantly male children in hot or warm climates. The primary symptom is intense ocular pruritus, often significant enough to affect activities of daily living. Clinical features differ from simple forms of allergic conjunctivitis in that they are more-or-less confined to the superior tarsus and limbus. There is also a risk of corneal involvement, which leads to irreversible vision loss in approximately 6% of patients. Right now, there is no standardized treatment protocol, and many of the currently available options are not effective in severe and recurrent cases. As such, it is imperative to understand this complex allergic immune response in order to identify future therapeutic targets. This review will focus on potential drug targets in VKC, with particular emphasis on immunomodulators and immunobiologic agents.

## 1. Introduction

**Figure 1:**
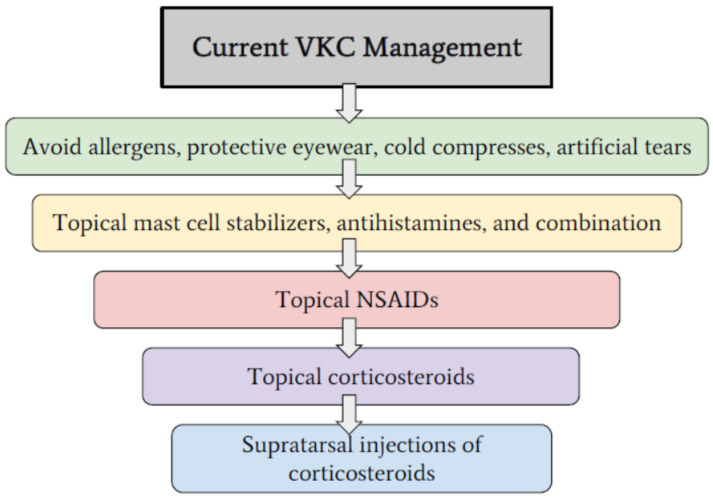
Breakdown of current stepwise approach to the clinical management of VKC and allergic ocular conditions.

**Figure 2:**
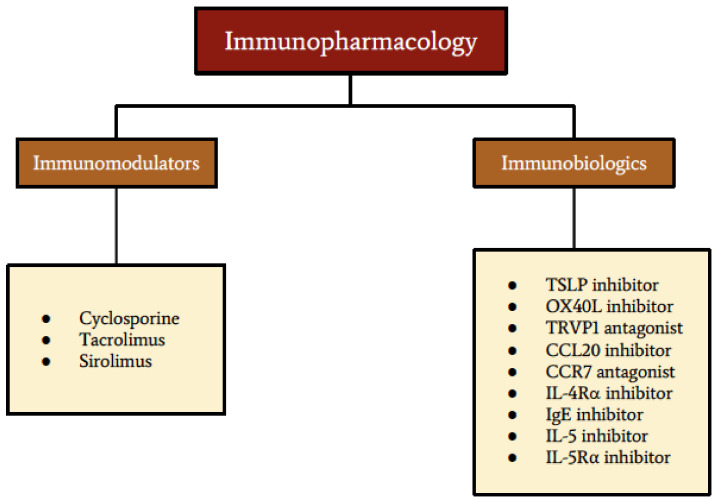
Summary of current and future immunopharmaceuticals for VKC.

Vernal keratoconjunctivitis (VKC) is a multifactorial Th2 cell-mediated ocular surface inflammatory condition that predominantly affects males, specifically children and young adults [1,2]. It is characterized by overexpression of mast cells, eosinophils, Th2 cells, cytokines, chemokines, and adhesion molecules. The hallmark features of VKC include conjunctival fibroproliferative lesions, limbal infiltration, conjunctival hyperemia, and corneal involvement [3,4,5,6]. The three forms of VKC based on the main anatomical site of the papillary reaction include: limbal, mixed, and palpebral VKC [7,8,9]. The tarsal form of VKC is common in temperate regions and is characterized by the presence of papillary hypertrophy on the upper palpebral conjunctiva [10,11]. The limbal form of VKC is characterized by the presence of gelatinous limbal papillary hyperplasia and is more common in warmer climates [12]. VKC usually resolves completely within 10 years of onset, but in some individuals, it could progress to atopic keratoconjunctivitis, particularly in the late teens and early twenties [2,9,10,13]. The perennial variant of VKC is more common in warmer climates, while the seasonal variant occurs mostly in temperate regions, with flare-ups occurring in the spring and summer [2]. However, this variation in presentation is dependent on the allergic disposition of the patient and the climate [9]. It is noteworthy that approximately 50% of patients with VKC do not have an immunoglobulin E (IgE)-dependent immune mechanism [13,14,15]. The most prominent clinical manifestations of VKC include papillae, perilimbal conjunctival hyperpigmentation, limbal infiltration, conjunctival hyperemia and chemosis, pruritus, persistent corneal defects, and vernal shield ulcer. Pharmacotherapy of VKC includes topical antihistamine/mast cell stabilizer combinations, mast cell stabilizers, non-steroidal anti-inflammatory drugs (NSAIDs), and corticosteroids (Figure 1) [16]. This review will provide a brief overview of immune and pathological mechanisms, clinical manifestations, and clinicopathological correlations of VKC. The focus of this review will be on potential drug targets in VKC, with particular focus on topical immunomodulators as well as future perspectives on immunobiologic agents and their targets (Figure 2).

## 2. Epidemiology and Disease Burden

VKC is a bilateral, seasonal allergic inflammation. Though the exacerbations are acute, the condition is chronic and classified as either perennial or seasonal. The perennial form, which represents 23% of cases, lasts throughout the year, whereas the seasonal variant occurs in the spring, as the name “vernal” suggests. There is a conversion rate of seasonal to perennial cases of 16% over 3 years [13].

VKC commonly affects children between the ages of 4–7 years old. A few cases report presentations as early as 5 months old and some as late as 38 years old. Typical resolution occurs post puberty [17,18]. VKC has a male predilection and is more common in dry, hot climates such as in the Mediterranean regions, Central and West Africa, Japan, India, the Middle East, and South America [19,20,21,22,23]. Allergic conjunctivitis, in all of its forms, affects 6–30% of the general population and about 30% of children [20].

VKC comprises 0.1–0.5% of ocular diseases in the developed world. In Europe, the prevalence is quite rare, ranging from 1.2–10.6 cases per 10,000 [24]. It affects males 3 to 4 times more frequently than females, with 80% of cases presented in children under 10 years of age [25,26,27]. In the event that VKC affects adults, the male to female ratio is equal [5,26]. The VKC-like disease is a subset of VKC, which affects post-pubescent or adult populations. While the clinical presentation is similar to the typical VKC that occurs in children, it often results in tarsal conjunctival fibrosis without the classic giant papillae formation and are also less likely to develop corneal infiltration [28,29].

The incidence of VKC rises in the warmer, drier climates in Africa, the Middle East, Latin America, and Asia. In Africa, it is reportedly as high as 37% and up to 7.3% in Ethiopia [30,31,32,33]. Additionally, numerous hospital referrals in Africa, the Middle East, and Asia are a result of VKC, and it is the main condition among children in general eye clinics. Corneal complications due to VKC range from 7% to 50% in patients presenting to hospital facilities in tropical regions [34,35]. Consequently, this accounts for a significant cause of school absences in the pediatric population [31].

Palpebral forms of VKC are more common in Europe and North/South America, while limbal forms are more prevalent in Africa and mixed forms in Asia [34,35]. Palpebral forms, constituting upper conjunctival cobblestone papillae, are more common in the United States and Europe, whereas mixed forms of VKC dominate in the African and Asian populations [25].

Regardless of the age or type of presentation, VKC can have a significant impact on quality of life, affecting day to day activities and schooling, as well as the potential normal development of psychological issues and relationships [25,26,36,37]. This poses a significant economic burden, as VKC restricts children and adults from performing their normal, daily activities and may result in serious, irreversible visual complications [14,38]. Without timely and effective diagnosis and treatment, 4.6% of patients with VKC suffer from a visual deficit [38,39].

## 3. Cells and Mediators

The immunopathogenesis and immunopathology of VKC involve Th2 cells, eosinophils, mast cells, cytokines, chemokines, and adhesion molecules. Mediators generated by Th2 cells, mast cells, and eosinophils exacerbate allergic inflammation at the ocular surface [40,41,42,43]. Additionally, dendritic cells (DC) resident in the conjunctiva play a prominent role in the immunopathogenesis of VKC [44]. Th2 cells are a subset of CD4^+^ T cells, which are derived from common lymphoid progenitor cells in the bone marrow. Cytokines derived from Th2 cells are involved in the immunopathological reactions of VKC. Interleukin-4 (IL-4), IL-5, and IL-13 are cytokines derived from mast cells and Th2 cells [45,46,47]. Adhesion molecules play a pivotal role in the recruitment of T cells to sites of allergic inflammation in the conjunctiva. Extravasation of Th2 cells into the site of allergic inflammation in the conjunctiva occurs in response to leukocyte function-associated antigen-1 (LFA-1)-intercellular adhesion molecule-1 (ICAM-1) interaction at the vascular endothelium [48]. ICAM-1 is upregulated on conjunctival fibroblasts and epithelium following an allergic reaction, and it enhances the influx of inflammatory cells such as eosinophil into the site of allergic inflammation on the ocular surface [43,49].

Epithelial cells and fibroblasts of the conjunctiva are non-immune cells that participate in the immunopathogenesis and immunopathology of VKC. Conjunctival epithelium can be activated to secrete cytokine, chemokine, and adhesion molecules that promote the influx of eosinophil and Th2 cells to the site of conjunctival inflammation [50]. Fibroblasts can be activated to produce mediators of inflammation. In VKC, IL-4 and IL-13 can activate corneal fibroblasts to secrete C-C Motif Chemokine Ligand 11 (CCL11), matrix metalloproteinase (MMP), ICAM-1, and CCL17. CCL11 mediates the recruitment of eosinophils, whereas CCL17 mediates the recruitment of Th2 cells [45,51,52,53,54]. Thus, the epithelial cells and fibroblasts of the cornea and conjunctiva participate in immunopathological processes that result in tissue damage and remodeling in allergic eye disease.

The connective tissue-type mast cells in the substantia propria of the conjunctiva play an important effector function in ocular allergy [55]. Activated conjunctival mast cells secrete histamine, lipid mediators, cytokines, chemokines, and growth factors. These mediators participate in the inflammatory reaction in VKC. Cytokines (IL-4, IL-5, TNF-α (tumor necrosis factor-alpha), and IL-13) and chemokines (CCL5, CCL11, and CCL17) are released by activated conjunctival mast cells [56]. Leukotrienes, prostaglandin, and platelet-activating factors (PAF) are lipid mediators released by activated and degranulated conjunctival mast cells. Leukotrienes are capable of inducing vasodilation and capillary permeability of conjunctival vessels, whereas prostaglandin D2 plays a role in inducing conjunctival vasodilation, intensifying the ocular itch sensation, and inducing the release of mucus from goblet cells [50,57,58]. PAF plays a major role in VKC. It mediates the chemotaxis of eosinophils to the site of conjunctival inflammation in VKC [57,59]. Histamine released by mast cells interact with H1 and H2 receptors (H1R and H2R) expressed on conjunctival epithelial cells, which results in the activation of conjunctival epithelial cells. Histamine-activated conjunctival epithelium secretes cytokine, chemokines, adhesion molecules, and MMP [35].

C-C chemokine receptor 6 (CCR6) is a chemokine receptor expressed on immature DC, effector/memory CD4^+^ T cells, and B cells [60]. C-C chemokine receptor 7 (CCR7), a receptor for CCL19 and CCL21, is responsible for directing the trafficking of mature DC to the regional lymph node [61]. CCR7 is expressed on naïve T cells, central memory T cells, and mature myeloid DC [62]. CCR6-CCL20 interactions are responsible for the chemotaxis of immature DC and effector/memory Th2 cells [60].

A few studies have demonstrated the role of thymic stromal lymphopoietin (TSLP) and OX40L in initiating the immunopathogenesis of allergic eye disease. Allergen-activated conjunctival epithelial cells upregulate the expression of TSLP, which in turn activates conventional DC in the conjunctiva to upregulate the expression of OX40L. TSLP-activated DC expressing CCR7 and OX40L traffic to the regional lymph node to promote Th2 cell polarization. Th2 cells participate in both immunopathogenesis and immunopathology of allergic processes in VKC [63,64].

Histamine, IL-4, TNF-α, and IL-13 interact with conjunctival fibroblasts to induce their activation, proliferation, the release of chemokines, and upregulation of ICAM-1 [65,66,67,68]. In VKC, IL-4 and TNF-α activated corneal fibroblasts upregulate the expression of ICAM-1 and secrete CCL17 and CCL11 [68]. CCL17 [69] and CCL11 [70] promote the recruitment of Th2 cells and eosinophils, respectively, whereas ICAM-1 promotes the infiltration of Th2 cells and eosinophil into the conjunctiva [46]. IL-5 is a cytokine produced by both Th2 cells and mast cells that plays a role in recruiting eosinophil to the site of allergic inflammation of the ocular surface in VKC [46]. It has been reported that patients with VKC have significantly high levels of TNF-α in the tear film, and this suggests the important role TNF-α plays in the immunopathology of VKC [71]. TNF-α, produced by activated conjunctival mast cells in the setting of allergic inflammation in VKC, is responsible for upregulating the expression of ICAM-1 [72] and ICAM-2 [48]. ICAM-1 upregulation on conjunctival and corneal fibroblasts plays a role in enhancing the influx of inflammatory cells, such as eosinophils, to the site of allergic inflammation [43,49].

It is of note that chemokines expressed by activated epithelial cells mediate the recruitment of Th2 cells and eosinophils to the ocular surface. MMPs break down the intercellular junction linking ocular surface epithelial cells and degrade the extracellular matrix. This facilitates the infiltration of immune cells into the subepithelial layer of the conjunctiva [35]. CCL5 is a chemokine produced by immune and non-immune cells that mediates the recruitment of eosinophil to the site of allergic inflammation of the conjunctiva [10]. Activated ocular surface epithelial cells express CCL11, which in turn creates a chemokine gradient that promotes the infiltration of eosinophils into the conjunctiva of patients with VKC [73]. It is noteworthy that eosinophil is responsible for the tissue remodeling and damage of the conjunctival tissue in VKC [10].

Eosinophil is a major innate effector cell that mediates type 2 immune responses. In allergic reactions, it has been demonstrated that eosinophils produce various mediators that exacerbate the chronic inflammation of the conjunctiva in VKC. Eosinophil-derived granules such as eosinophil cationic protein (ECP) and eosinophil major basic protein (EMBP) can disrupt the barrier function of the epithelium of the ocular surface. EMBP is a major mediator produced by activated eosinophils that has been shown to be toxic to the epithelial cells of the ocular surface [57,74,75]. MMP9 secreted by activated and degranulated eosinophils degrade the extracellular matrix, which leads to ocular surface remodeling in patients with VKC [11,76]. Thus, MMP and eosinophil-derived granule proteins participate in promoting the access of immune cells and mediators to the corneal stroma through the compromised barrier function of the cornea [42].

## 4. Clinical Signs and Symptoms

The most commonly reported symptom is ocular pruritus that is often intense enough to affect daily activities. Photophobia, foreign body sensation, and tearing can also occur and, if severe, may indicate corneal involvement. Mucoid discharge and tearing which results in eyelids being stuck together on awakening is common to VKC [35]. The immune response in VKC alters the normal cytology of the conjunctiva, resulting in mast cell and eosinophil aggregation, which is otherwise absent in a healthy eye. The presence of these cells is proportional to the severity of clinical signs and symptoms that are experienced [77,78]. Unlike the many more common forms of allergic conjunctivitis, VKC is unique in that it has the potential to lead to sight-threatening complications [26].

Similar to conjunctivitis, the hallmark clinical feature of VKC is papillae. It is distinguished from other types of allergic conjunctivitis in that it predominantly affects the upper tarsus and limbus [35]. These papillae are larger, up to 5 mm in size, with flattened tops, manifesting clinically as “cobblestone” papillae. These giant papillae contain cells that are commonly implicated in allergic conditions, such as mast cells, eosinophils, and neutrophils, among a fibrovascular center surrounded by conjunctival cells [79]. The subsequent inflammation that results can also lead to diffuse conjunctival hyperemia. As the common site of clinical features is confined to the superior tarsus, pseudomembrane formation may be evident in that location in response to the mucoid discharge being exposed to heat. This is known as the Maxwell Lyon sign [17].

Few cases have described additional superior conjunctival features arising from a granulomatous reaction known as the Splendore-Hoeppli phenomenon. This nonspecific sign is indicative of eosinophilic infiltration within a granulomatous reaction and appears clinically as yellow, subconjunctival nodules that are accompanied by tortuous vasculature [80].

**Figure 3:**
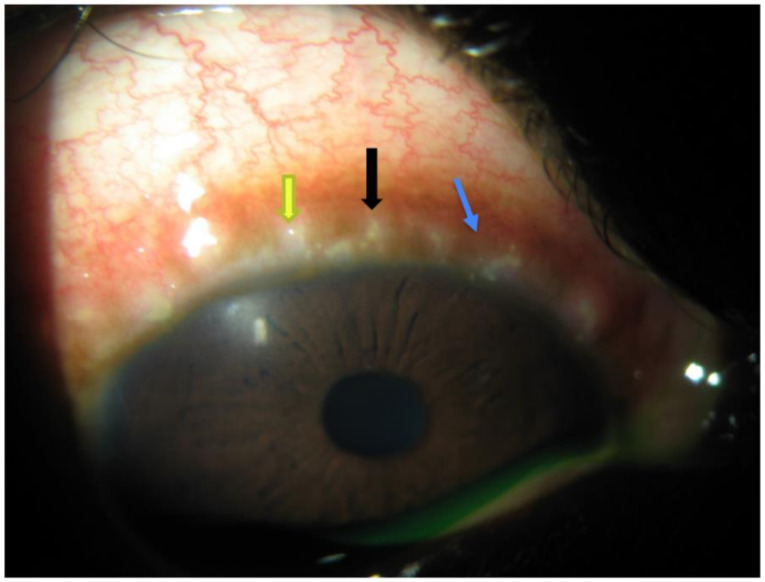
Limbal form of VKC characterized by the presence of gelatinous limbal papillary hyperplasia (black arrow), Horner-Trantas dots (yellow arrow), and perilimbal conjunctival hyperpigmentation (blue arrow).

VKC can also affect the limbus and appear as a round, gelatinous papillae with a focal white center that is composed of eosinophils and epithelial cells. This finding is called Horner-Trantas dots (Figure 3) and can lead to the development of corneal neovascularization [17]. Additional corneal involvement includes pseudogerontoxon, which appears as a grayish-white deposition of lipid in the peripheral, superficial stroma [81].

Corneal injury results from the mechanical force between the giant conjunctival papillae and corneal epithelium that may cause shield ulcers. Additionally, this process may yield the release of inflammatory mediators from eosinophils and mast cells, leading to fibrin plaque development and poor wound healing [42,52,82,83]. In severe cases, shield ulcers form, which begin as punctate corneal erosions that progress to larger erosions that penetrate Bowman’s layer. Plaque formation over the site of ulceration, made up of fibrin and mucous, can delay corneal healing and contribute to neovascularization [29,84]. Shield ulcers offer a means of microbial invasion and subsequent keratitis. This is a somewhat common complication of VKC, present in 9–10% of cases. The two most common bacterial culprits are *Staphylococcus epidermis* and *Streptococcus pneumoniae*. *Corynebacterium*, *Neisseria meningitides*, *Klebsiella pneumoniae*, and *Brevibacterium* species have also been reported, as well as fungal infections due to *Aspergillus* species [85]. If left untreated, VKC can result in irreversible vision loss [86]. A permanent reduction in visual acuity is documented in up to 6% of affected patients [42,83]. There is no standardized treatment protocol [87]. Further complications include keratoconus, deprivational amblyopia, and corneal perforation [17]. Limbal forms can lead to stem cell deficiency that can further harm the ocular surface and impact vision, resulting in chronic ocular surface inflammation, corneal epithelial defects, and the growth of conjunctival epithelium onto the corneal surface [35]. A secondary ptosis is possible, likely attributable to the presence of giant papillae of the upper lid, chronic eye rubbing, or inflammatory damage to the levator palpebrae superioris muscle [26].

Vision loss from VKC is reported in up to 30% of cases, occurring secondary to shield ulcer formation, corneal neovascularization, or the development of keratoconus. Indirect causes of vision loss may also result from chronic corticosteroid treatment leading to cataracts or glaucoma [88]. Other than the ocular manifestations of VKC, there is some risk of systemic involvement in the form of rhinitis or the development of asthma following the resolution of ocular symptoms [26].

## 5. Clinicopathological Correlations

Immune cells, fibroblasts, epithelial cells, cytokine, chemokines, and adhesion molecules are responsible for the clinical expressions of VKC in the immunopathology of VKC, such clinical expressions include conjunctival hyperemia and chemosis, papillae, itch, perilimbal conjunctival hyperpigmentation, pseudogerontoxon, Horner-Trantas dot, persistent corneal defect, and shield ulcer [89,90,91,92].

Histamine interacts with H1 receptors (H1R) and H2R expressed on conjunctival fibroblasts to induce their activation, proliferation, and production of extracellular matrix (ECM) [65]. In addition to histamine, IL-4 and IL-13 interact with their respective receptors on conjunctival fibroblasts to induce both the proliferation of conjunctival fibroblasts and production of excessive amounts of ECM [66,67]. Deposition of excessive amounts of ECM in the conjunctival tissue gives rise to conjunctival fibroproliferative lesions, such as giant tarsal papillae and limbal gelatinous hyperplasia. It is important to note that increased synthesis of ECM by activated conjunctival fibroblasts, as well as the subsequent deposition and accumulation of ECM in the conjunctiva, is responsible for the extracellular matrix hyperplasia that manifests in giant papillae and limbal papillary hyperplasia [52,93]. Because IL-4, IL-5, IL-13, and histamine play a major role in the immunopathology of VKC, they are considered important therapeutic targets in VKC.

Histamine interacts with histamine receptor 1 (H1R) and H2R on conjunctival blood vessels to induce conjunctival vasodilation and capillary leakage, which manifests as conjunctival hyperemia and chemosis, respectively. The ocular itch sensation is due to the interaction between histamine and H1R on conjunctival sensory nerve fibers [51,94]. Leukotriene is responsible for conjunctival hyperemia and chemosis because it can induce conjunctival vasodilation and vasopermeability, respectively [95,96]. Additionally, leukotrienes can increase the secretion of mucus from conjunctival goblet cells. A study demonstrated that resolving D1 and E1 promotes the resolution of inflammation by reducing leukotriene-mediated secretion of mucus from goblet cells [97]. Prostaglandin D2 can cause conjunctival hyperemia by inducing conjunctival vasodilation. It is responsible for mucus production via its interaction with conjunctival goblet cells. Prostaglandin D2 has also been shown to intensify pruritus [50,57,58,98]. IL-5 activates and recruits eosinophils, whereas IL-13 induces mucus production in conjunctival goblet cells [51,99,100].

Perilimbal bulbar conjunctival hyperpigmentation is due to deposition of melanin in the perilimbal area of the bulbar conjunctiva. Histamine [101] and stem cell factor [102] released by cytokine-activated conjunctival fibroblasts [103] interact with their respective receptors on conjunctival melanocytes to induce the release of excessive amounts of melanin, which are deposited in the perilimbal bulbar conjunctiva [104,105]. Pseudogerontoxon is a corneal finding present in patients with VKC. It is due to chronic limbal vasopermeability with its associated accumulation of lipids in the anterior stromal cornea [10,106,107]. Horner-Trantas dots and gelatinous limbal infiltrates are hallmark features of limbal VKC. Horner-Trantas dots are composed of clumps of necrotic eosinophils, epithelial cells, and neutrophils [94,108,109]. Persistent corneal epithelial defects in VKC are due to mediators released by histamine-activated epithelial cells and eosinophil-derived granules. Furthermore, damage to limbal stem cells has a role to play in delayed re-epithelialization of corneal epithelium. Similar to a persistent corneal epithelial defect, a corneal shield ulcer is due to a combination of both mechanical and immune factors. The chronic mechanical abrasion of the corneal epithelium by giant papillae on the superior tarsal conjunctiva during blink action has been shown to activate corneal epithelial cells to overexpress pro-inflammatory mediators. In immune-mediated mechanisms of corneal shield ulcer, MMP secreted by activated epithelial cells, eosinophils, and corneal fibroblasts play a pivotal role in the pathogenesis [12,83,110,111,112]. Additionally, eosinophil-derived granules released by recruited eosinophils participate in the disruption of corneal barrier function and break down of the corneal stromal matrix [12,113,114]. The degradation of the corneal stromal matrix manifests as a vernal shield ulcer, which is more common in patients with the tarsal form of VKC.

## 6. Management

The primary mode of treatment for all allergic diseases is avoidance of known allergens. However, this is not always practical as it may be difficult to avoid certain allergens specific to a region. HEPA filters have proven to be an effective environmental filtration tool in allergic rhinitis, but have added little or no benefit to the associated ocular symptoms. Protecting the eye from environmental allergies with wraparound sunglasses can reduce contact. Other non-pharmacologic care includes the use of cold compresses, which may alleviate ocular symptoms due to the vasoconstrictive effect, limiting the degree of hyperemia and edema [115]. Artificial tears may aid in flushing the tear film of allergens and inflammatory cells [116,117].

## 7. Therapeutic Targets for Pharmacotherapy

Pharmacologic treatment options include acute and chronic therapy depending on the variable and recurrent nature of VKC presentations. In mild forms of the disease, topical mast cell stabilizers, antihistamines, and combination formulations are sufficient for symptomatic relief but are inadequate in targeting the depth, complexity, and chronicity of this inflammatory condition [5,16,37,118].

Mast cell stabilizers inhibit mast cell degranulation and cytokine release, thus suppressing the allergic response. These drugs include 2% and 4% sodium cromoglicate (DSCG (disodium cromoglycate), cromolyn), nedocromil sodium 2%, spaglumic acid 4%, and lodoxamide tromethamine 0.1%. Lodoxamide tromethamine has the added effect of inhibiting eosinophil degranulation and was found to be the most effective of the antiallergic therapeutic agents. Nedocromil also has additional inhibitive effects on neutrophils, macrophages, monocytes, and platelets. When using mast cell stabilizers for VKC, majority of them are often administered 4–6 times per day depending on symptom onset and duration [119,120,121,122,123,124].

Topical antihistamines block histamine release from the conjunctival mast cells. H1 blockers are most commonly used due to their longer duration and mild side effects. These drugs include levocabastine hydrochloride 0.5% and emedastine 0.05%. Between these two agents, studies reported higher efficacy with emedastine [125,126,127].

Combination mast cell stabilizers and antihistamines offer the benefit of both acute and chronic therapy due to a more rapid onset of action and long-term therapeutic benefits. Topical combination or dual-acting therapies include alcaftadine (0.25%), azelastine (0.05%), bepotastine besilate (1.5%), epinastine (0.05%), ketotifen (0.025%), and olopatadine (0.1%, 0.2%, 0.7%) [5]. In addition to the antiallergic effect of ketotifen and olopatadine, these drugs also possess anti-inflammatory properties due to their effect on tumor necrosis factor-alpha from conjunctival epithelial cells and eosinophils [17,89,124].

Topical nonsteroidal anti-inflammatory drug (NSAID) targets the inflammatory pathway at the level of prostaglandin E2 and I2 by inhibiting their release. These agents include ketorolac tromethamine (0.5% and 0.4%), bromfenac sodium (0.09% and 0.07%), and diclofenac sodium (0.1%). Due to the well-known side effects of corneal toxicity with prolonged topical NSAID use, caution is exercised in VKC with corneal involvement. These drugs are efficacious in symptomatic relief but are not beneficial in the resolution of corneal wound healing or reducing giant papillae [128].

In more moderate cases, topical corticosteroids may be utilized to suppress the inflammatory response, though their risk of side effects prohibit long-term use. Though corticosteroids may be effective, chronic use carries the risk of cataract formation, glaucoma, and secondary microbial keratitis [5,25]. Corticosteroids can be administered topically or ocularly through a supratarsal injection. In severely symptomatic cases with a high degree of inflammation, especially in cases that do not respond to the aforementioned therapies, steroids can be efficacious. In such cases, the low potency or soft steroids, are used initially. These include fluorometholone acetate 0.1%, loteprednol etabonate 0.5%, and rimexolone 1%. If this subset fails, those with a higher potency may be used, such as prednisolone acetate 1%, dexamethasone sodium phosphate 0.1%, or difluprednate 0.05%. While these agents are very effective anti-inflammatory agents, they are reserved for severe or unresponsive cases due to the risk of side effects with long-term use, such as cataract formation and glaucoma [5].

Severe, refractory cases of VKC that are unresponsive to topical therapy may respond to targeted, supratarsal steroidal injections aimed to suppress the inflammatory response. Triamcinolone acetonide (10.5–20 mg), dexamethasone sodium phosphate (2 mg), and hydrocortisone sodium succinate (50 mg) are injected into the superior tarsal border between the conjunctiva and Muller’s muscle. This can aid in a significant reduction in the size of both papillae and shield ulcers, as well as resolution of lid edema, conjunctival chemosis and discharge, and Horner-Trantas dots. Though effective, these injections may lead to hazardous effects including elevated intraocular pressure and glaucoma, eyelid necrosis, and linear subcutaneous fat atrophy [129,130,131,132].

## 8. Therapeutic Targets for Immunomodulators

Topical immunomodulators have a good safety profile and are effective in the treatment of severe forms of VKC. Calcineurin inhibitors, such as cyclosporine and tacrolimus, are immune modulators that work to block IL-2 mediated Th2 lymphocyte proliferation, an important component in the pathogenesis of VKC. The VEKTIS trial, which evaluated the efficacy and safety profile of cyclosporine A (CsA) in children and adolescents with severe VKC, concluded that 1 month of treatment offered a significant therapeutic benefit when compared to subjects who received vehicle alone [37].

CsA is an insoluble and lipophilic cationic emulsion that inhibits Th2 proliferation and IL-2 production [133]. Thus, CsA leads to reduced levels of IL-2 and Th2 derived cytokines, as well as decreased activation of eosinophils [99] and mast cells [134]. Additional effects of CsA include blocking IL-5, a cytokine that is implicated in the activation and recruitment of eosinophil. This further inhibits the recruitment of eosinophils and enhanced survival and release of eosinophilic granule contents [99]. This mechanism of action directly reduces the number of immune cells present at the ocular surface. In this manner, CsA is able to modulate the immune response and reduce the signs and symptoms of VKC with limited side effects [118,135,136,137,138]. CsA has been effective in VKC treatment at various concentrations, but is available commercially in only 0.05% and 0.01% concentrations. Studies evaluating the efficacy of topical ophthalmic CsA at a 0.05% concentration reported decreased levels of IL-4, IL-5, IL-17A, TNF-alpha, interferon-gamma, and eotaxin in the tear film of treated patients, as well as a reduction in the density of inflammatory cells within the conjunctiva [79,90,139]. The topical ophthalmic CsA 2% concentration has been proven effective in improving the signs and symptoms of VKC, whereas the 1% concentration was not as potent. In fact, when compared to topical ophthalmic dexamethasone 0.1%, both displayed comparable efficacy with the added benefit of safety in 2% CsA subjects. At lower doses of topical ophthalmic CsA, such as the 0.05% and 0.1% concentrations, it was found that topical corticosteroids were superior in controlling inflammation. However, the lower concentrations are safe and effective for chronic use to limit recurrences and keep the VKC at bay [35]. Though treatment of VKC with CsA remains off-label, it is an effective option with four times daily dosing and can be used long term for severe and chronic VKC cases with a better safety profile than corticosteroids [140]. It is of note that Gokhale and colleagues reported success with oral administration of cyclosporine in the management of sight-threatening VKC [141].

Tacrolimus is another calcineurin inhibitor that works similarly, downregulating the inflammatory response. Topical tacrolimus is similar in functionality to CsA and also beneficial in VKC treatment. However, it is more potent, making it more likely for patients to develop opportunistic infections [35,142,143]. Tacrolimus comes in various concentrations, with 0.01% and 0.005% ophthalmic drops administered four times daily, reportedly effective for refractory VKC [142,144]. Side effects are also minimal, with ocular irritation being the most commonly reported [143,145,146]. A study, comparing the use of 0.1% tacrolimus ophthalmic ointment dosed twice a day versus 2% CsA eye drops four times a day for the treatment of VKC, did not find a statistically significant difference between the two groups. When compared to each other, both 0.1% tacrolimus ophthalmic ointment and 2% cyclosporine ophthalmic drops evaluated at 4 weeks and 8 weeks of treatment were similarly effective against the signs and symptoms of VKC. Furthermore, the safety profile and side effects were comparable and minimal [147].

VKC, like most types of allergic conjunctivitis, has an IgE-mediated component, allowing the potential for immunotherapeutic targets to quell the response. It is a complex mechanism that initiates an inflammatory response mediated by Th2 cells, eosinophils, and Th2-derived cytokines [148]. Immunotherapy works to downregulate the Th2-mediated immune response while increasing the regulatory T cell response to reduce the response to exposure. It not only offers symptomatic relief during the course of treatment, but also relief in greater durations following the completion of the treatment course [149]. At this time, both subcutaneous and sublingual therapies are available. Both are recommended for the treatment of allergic rhinoconjunctivitis but have not been thoroughly investigated for VKC [149,150,151]. Furthermore, oral immunomodulators are also not typically used for VKC, even in severe cases, despite reports that oral cyclosporine and intravenous immunoglobulin therapies were effective [141,152]. Subcutaneous immunotherapy, both pre-seasonal and perennial treatments, has been shown to offer a significant reduction in mediating the ocular symptoms associated with allergic disease [153]. More recently, sublingual immunotherapy is also available, in either tablet or drop forms. With most allergens, these formulations were effective in relieving the ocular symptoms of itching, tearing, and redness [154].

Patients receiving specific immunotherapy, as recommended by the World Health Organization (WHO), had an improvement in symptoms of conjunctivitis, rhinitis, and asthma. This therapy works by administering high doses of allergen via subcutaneous or sublingual routes. An immune response is initiated, favoring the generation of Th1 cells and regulatory T cells. Consequently, the release of IL-10 and transforming growth factor-beta (TGF-β) by regulatory T cells work to suppress the Th2-mediated allergic response [155,156]. When compared, the subcutaneous immunotherapeutic response was more effective than the sublingual treatment route, especially in the adult population [157].

Sirolimus or rapamycin, is a significantly more potent immunosuppressant than cyclosporine, with an even greater safety profile than both cyclosporine and tacrolimus. Sirolimus is a macrolide antibiotic that is able to penetrate the ocular surface and tissues, reaching a high concentration within the aqueous humor. It inhibits mechanistic targeting of rapamycin complex 1 (mTORC1), which regulates protein synthesis, cell growth/proliferation and metabolism, and stress response [158]. Effects of sirolimus are mediated through phosphorylation of p70S6 kinases. It subsequently decreases VEGF production, allowing for its anti-angiogenic properties [159,160]. Corneal neovascularization, a well-known sight-threatening complication of VKC, arises from the inflammatory response that allows cytokine infiltration within the ocular tissue to cause tissue necrosis and angiogenesis. Sirolimus thus suppresses the G1-to-S phase transition of T cells, blocking IL-2, thus inhibiting angiogenesis and cell proliferation, which can markedly reduce the inflammatory response related to allergic ocular conditions including VKC [161]. Sirolimus has been shown to yield potent anti-proliferative, anti-inflammatory, and immunosuppressive properties. This is due to its effect on CD4^+^ T cells, regulatory T cells, DC, and myeloid-derived suppressor cells. While its insolubility and low bioavailability have limited its ophthalmic use, its documented immunosuppressive effect with limited side effects warrant further modifications and studies for use in ocular allergy. Wang et al. provided evidence that when administered through a nano-micelle ophthalmic solution, rapamycin was able to effectively work on the corneal surface to prevent corneal-allograft rejection [162].

Allergic conditions such as rhinitis, asthma, and conjunctivitis, are able to be mediated through allergen-specific immunotherapies. It is an effective and under-utilized treatment approach that limits the various symptoms experienced with allergic conditions, as well as recurrences in the long term [149,163,164,165,166,167]. The goal of treatment is to achieve a balance between the Th2 and Th1 responses. IL-10 and TGF-β, produced by these regulatory T cells, combat the inflammatory response. IgE antibody levels are subsequently reduced, limiting the release of inflammatory cytokines from mast cells, eosinophils, and T cells [149,167,168]. Although the mechanisms of VKC are complex and multifactorial, it is well established that Th2-derived cytokines are implicated in the signs and symptoms, warranting further investigation of targeted immunotherapy as a viable treatment option.

## 9. Therapeutic Targets for Immunobiologics

Immune mediators and cells play a pivotal role in mediating the immune and pathological mechanisms that cause tissue damage and remodeling in VKC. Cytokines, chemokines, adhesion molecules, and histamine, as well as other mediators, participate in the immunopathological mechanisms that generate the clinical expressions of VKC. Targeting these mediators with immunobiologics could be beneficial in ameliorating inflammation and damage to the ocular surface. Additionally, downregulating the immune response mediated by activated eosinophils, mast cells, and Th2 cells is beneficial. However, targeting the signaling pathways and molecular interactions that enhance the recruitment, survival, and activation of these key players of VKC’s immunopathology is necessary.

It is of note that both TSLP and OX40L are involved in the process of initiating the Th2-mediated immune response in ocular allergy. Zheng et al. [169] have reported the overexpression of TSLP by the conjunctival epithelial cells of patients with VKC. Targeted therapy aimed at inhibiting the upregulation of TSLP and OX40L could be beneficial in attenuating the initial stages of the allergic pathological process. The use of soluble TSLP receptor immunoglobulin, which has been demonstrated to be effective in downregulating the expression of costimulatory molecules (CD40, CD80, and CD86) on pulmonary DC, could prove effective in attenuating TSLP-induced activation of conventional DC in the conjunctivas [170] of patients with VKC.

Mast cells and myeloid DC in the conjunctiva express OX40L, OX40-OX40L interaction participates in the allergic inflammatory process [171]. A study demonstrated that blockage of OX40L resulted in reduced infiltration of eosinophils and Th2 cells (effector and memory Th2 cells) into the conjunctiva and consequentially resulted in reduced levels of Th2 cell-derived cytokines. Thus, the inhibition of OX40L resulted in the reduced capability of TSLP-activated conventional DC to promote Th2-induced inflammation [172]. Furthermore, a monoclonal antibody, which works against OX40L, can attenuate the allergic inflammatory process [171]. These studies highlight the potential role an inhibitor of the OX40L-OX40 interaction could have in attenuating Th2-mediated inflammation [171,172]. Because TSLP-activated myeloid DC upregulate the expression of OX40L, there is a likelihood that immunobiological agents that block TSLP and/or OX40L activity may be a beneficial targeted therapeutic in VKC. Inhibitors of TSLP-TSLP receptors and OX40-OX40L pathways could be considered potential immunobiologic agents in VKC.

Conjunctival epithelial cells express transient receptor potential vanilloid receptor-1 (TRVP1). Sensory neurons in the conjunctiva express histamine receptor-1 and TRVP1 [173]. The activation of these receptors by histamine is responsible for the ocular itch experienced in individuals with allergic conjunctivitis. Shim et al. [174] demonstrated that histamine-induced itching through the stimulation of phospholipase A2 and lipoxygenase involves the activation of TRPV1 by histamine. Kwon et al. reported that intraperitoneal injection of a TRPV1 antagonist attenuated the clinical manifestations of allergic conjunctivitis in a murine model [175]. This could be attributed to reduced infiltration of Th2 cells, as it has been reported that TRPV1 antagonist has an inhibitory effect on TCR signaling pathways and activation of CD4^+^ T cells. It is of note that TRPV1 and CD4 are expressed on CD4^+^ T cells [176]. Huang et al. [177] demonstrated that TRPV1 inhibitors attenuated histamine-dependent pruritus in allergic conjunctivitis. As such, inhibition of TRPV1 could be beneficial in ameliorating the itch sensation in ocular allergy.

It is noteworthy that chemokines interact with CCR6 and CCR7 to mediate the chemotaxis of DC, which participate in the allergen-driven immune response [60,61]. Chung et al. [178] reported that CCR6–CCL20 interaction plays a role in the allergen-driven Th2 immune response, and also that inhibition of CCL20 could ameliorate the Th2-driven allergic inflammation in the conjunctiva. A study by Schlereth et al. demonstrated that inhibition of CCR7 with a topical CCR7 antagonist ameliorated the clinical expressions of allergic conjunctivitis in immunized mice [179]. Thus, the inhibition of CCR7 and CCR6 in patients with VKC can ameliorate Th2-mediated immune and pathological mechanisms. CCL20 is expressed by activated conjunctival epithelial cells in VKC, and inhibition of CCL20 results in a reduced influx of CCR6-expressing memory T cells as well as inhibit the migration of immature DC [73]. Thus, CCL20 inhibition could be therapeutically beneficial for individuals with VKC.

Dupilumab is a monoclonal antibody approved in patients 12 years and up who suffer from severe, refractory cases of atopic dermatitis in which features of allergic conjunctivitis often coexist [180,181,182,183,184,185,186,187,188]. Dupilumab is a human monoclonal antibody against interleukin-4 receptor alpha, and as such, it has been shown that dupilumab can inhibit signaling of IL-4 and IL-13. IL-4 and IL-13 are important Th2-derived cytokines [189,190] since they play a role in the pathogenesis of giant papillae in the conjunctiva of patients with chronic ocular allergy. Dupilumab is typically administered via subcutaneous injection at a dose of 300 mg every 2 weeks when patients are not responsive to topical or oral treatments [191]. Fukuda and colleagues reported that dupilumab was effective in treating giant papillae on the palpebral conjunctiva of two patients with atopic keratoconjunctivitis, and as such, they suggested the potential benefits of using dupilumab as an immunobiologic agent in individuals with severe forms of chronic ocular allergy [192]. However, there have been reports of dupilumab-associated ocular surface disease in patients with atopic dermatitis [193,194,195,196]. There is currently a multi-center, double-masked, randomized, and placebo-controlled, parallel-group study that is evaluating the safety and efficacy of dupilumab in the treatment of signs and symptoms of individuals with atopic keratoconjunctivitis (NCT04296864). It is often used in adjunct therapies to treat eosinophilic diseases, thus showing its potential effectiveness in VKC.

Omalizumab is a recombinant humanized monoclonal antibody that reduces the availability of free IgE. This is accomplished by omalizumab binding to the Fc portion of IgE, thus preventing IgE from binding to Fc Epsilon Receptor I (FcεRI) on mast cells. Additionally, omalizumab can dissociate IgE from the IgE–FcεRI complex on primed mast cells [197]. As such, omalizumab can attenuate IgE-mediated responses by depleting circulating free IgE [198]. Beck et al. [199] reported that omalizumab could induce the downregulation of FcεRI expression on mast cells. Studies have also demonstrated that there is a significant reduction in both nasal and ocular symptoms when compared to placebo, beginning at 12 weeks of use. While case reports on using omalizumab to treat VKC showed promising results, it remains an off-label use for allergic eye diseases [198,200,201,202,203]. Further research is required to determine the dosage and exact administration that would yield the greatest therapeutic benefit [29,198,201]. Because there is an IgE-mediated mechanism in VKC, there is potential for omalizumab to be effective in the management of severe forms of VKC.

In contrast to other types of allergic conjunctivitis, VKC is unique in that it is a severe form of ocular allergy that is a result of massive eosinophil activation and infiltration [204]. It is of note that eosinophils play a major role in the immunopathology of VKC, as eosinophil-derived granules are very toxic to ocular surface cells. Corneal involvement, such as punctate epitheliopathy, ulcers, and plaque formation, that may complicate VKC is attributed to the toxicity associated with this high degree of eosinophilic reaction [205,206]. Diminishing this eosinophilic activation that is responsible for the clinical manifestations of VKC can be achieved by targeting IL-5, a cytokine that is involved in eosinophil activation, recruitment, maturation, and survival [207]. Targeting IL-5 and its receptor has been demonstrated to be beneficial in the management of eosinophil-mediated allergic disorders, such as asthma. Targeting IL-5 prevents it from accessing IL-5-receptor-alpha-chain (IL-5Rα) on eosinophils, whereas targeting IL-5Rα on eosinophils prevents the interaction of IL-5 with its receptor. This targeted therapy results in reduced eosinophilia. Thus, targeting eosinophil by blocking the IL-5-IL-5Rα interaction necessary for the maturation, activation, recruitment, and survival of eosinophils could be a potential therapeutic strategy in VKC. This is achieved through the development of three clinical immunotherapeutic agents: mepolizumab, reslizumab, and benralizumab. Mepolizumab is an IgG1-type antibody that binds to soluble IL-5, subsequently inhibiting its binding to IL-5Rα and impacting eosinophil activation. As of this moment, this drug, administered intravenously or subcutaneously, has been studied for its adjunctive use in asthma [208,209,210]. Research has indicated that mepolizumab treatment reduced the presence of eosinophils in the blood in a dose-dependent manner. Further research is warranted to determine the optimal dosage and long-term effects of anti-IL-5 antibody concentrations [210]. Reslizumab works similarly, but it is an IgG4-type monoclonal antibody. Studies have indicated similar efficacy of this drug in decreasing eosinophil levels in associated asthmatic conditions when administered through intravenous routes, which is also dose-dependent. In both drugs, an increase in serum IL-5 levels following treatment was observed, requiring further study on its long-term efficacy [210,211,212,213]. In contrast to the aforementioned drugs, benralizumab is an IgG1-type monoclonal antibody against IL-5Rα. As such, it binds directly to IL-5Rα on both eosinophils and basophils, prohibiting IL-5 binding to IL-5Rα. Additionally, it is an effective cytotoxic antibody due to its ability to complete target-cell depletion through interaction with FcyRIIIa receptors on natural killer cells [214,215]. Benralizumab has been FDA-approved as an IV adjunct therapy for asthma in children over the age of 12 due to studies indicating its effect in eosinophil and basophil serum depletion. Furthermore, a reduction in bone marrow eosinophil levels was also observed. While these drugs are utilized in the treatment of eosinophilic asthma, further investigation is necessary to determine optimal dosing as well as understanding the long-term efficacy and outcomes [210]. However, these drugs are significant because they may offer potential therapeutic benefits in other types of eosinophil-mediated conditions, such as VKC.

## 10. Conclusions

It is well established that VKC is a complex disease entity affecting many populations with the potential for irreversible vision loss and a significant reduction in quality of life. However, there remains no standard treatment protocol or consensus on treatment, particularly in severe and refractory cases of VKC. Currently, there is significant research that has offered insight into the specific, unique cellular and molecular aspects of the immunopathogenesis and immunopathology of VKC, leading to potential therapeutic targets for the immunomodulators (Table 1) and immunobiologics (Table 2) discussed in this comprehensive review. Targeting specific molecules, such as TSLP and OX40L, has been shown to attenuate the Th2-mediated inflammatory process in ocular allergy. The use of monoclonal antibodies to target specific cytokines, such as IL-5, involved in the pathological process of VKC could be beneficial in ameliorating the tissue inflammation and damage mediated by activated eosinophils in VKC. Current and future research should continue to focus on developing immunopharmacological agents that would be beneficial to individuals with VKC.

## Figures and Tables

**Table 1:** Summary of ocular therapeutic effects of immunomodulators.

Immunomodulator	Mechanism	Studied Ocular Therapeutic Effects
Cyclosporine	Calcineurin inhibitor	Decreases levels of IL-4, IL-5, IL-17A, TNF-alpha, interferon gamma, and eotaxin in the tear film, as well as reduces density of inflammatory cells within the conjunctiva, thereby effectively treating VKC [79,92,139].
Tacrolimus	Calcineurin inhibitor	Effective in refractory cases of VKC [140,141,142,147].
Sirolimus (rapamycin)	Inhibits mTORC1	Blocks IL-2 and inflammatory effects on the ocular surface, but it is not yet established in VKC treatment [158,161].

**Table 2:** Summary of immunobiologics and potential therapeutic mechanisms against VKC.

Immunobiologic Target	Mechanism of Therapy
TSLP inhibitor	Inhibition of TSLP expression by conjunctival epithelial cells in patients with VKC can downregulate the expression of costimulatory molecules (CD40, CD80, and CD86) on DC. This attenuates the Th2-mediated immune response in VKC [169,170].
OX40 inhibitor	Inhibition of OX40L expression by myeloid DC and mast cells can reduce the infiltration of eosinophils and Th2 cells into the conjunctiva. This attenuates the Th2-mediated inflammation in VKC [171,172].
TRVP1 antagonist	TRPV1 antagonist has an inhibitory effect on TCR signaling pathways and activation of CD4+T cells [176]. Blocking the expression of TRVP1 by conjunctival epithelial cells attenuates histamine-dependent pruritus in allergic conjunctivitis [177].
CCL20 inhibitor	Blocking CCR6-CCL20 interaction via inhibition of CCL20 expression can ameliorate Th2-driven allergic inflammation in the conjunctiva of patients with VKC [73].
CCR7 antagonist	CCR7 antagonists inhibit the migration of mature DC to the lymph node, resulting in amelioration of Th2-driven immunopathological mechanisms [179].
IL-4Rα inhibitor	Dupilumab is a monoclonal antibody against interleukin-4 receptor alpha, which can inhibit the signaling of IL-4 and IL-13 [189,190].
IgE inhibitor	Omalizumab is a monoclonal antibody that reduces the availability of free IgE and induces the downregulation of FcεRI expression on mast cells [197,198,199].
IL-5 inhibitor	Mepolizumab is an IgG1-type monoclonal antibody that binds to soluble IL-5, blocking IL5 from binding to IL-5Rα expressed on eosinophils. This results in reduced activation and recruitment of eosinophils [208,209,210].
IL-5 inhibitor	Reslizumab is an IgG4-type monoclonal antibody against IL-5, which results in a reduced level of eosinophils [210,211,212,213].
IL-5Rα inhibitor	Benralizumab is an IgG1-type monoclonal antibody against IL-5Rα on eosinophils that inhibits IL-5 from binding to its receptors on eosinophils, resulting in decreased activation and recruitment of eosinophils [214,215].

## Data Availability

Data sharing not applicable.
